# TRIM11 promotes cell proliferation of non‐small cell lung cancer through the inhibition of ferroptosis by AMPK

**DOI:** 10.1111/crj.13675

**Published:** 2023-08-21

**Authors:** Zheng Liang, Jian Li, Guoliang Zhang, Menghui Chen

**Affiliations:** ^1^ Department of Cardiothoracic Surgery The Third Hospital of Shijiazhuang Shijiazhuang China; ^2^ Department of Orthopedics, The Hospital 731 China Aerospace Science and Industry Group Beijing China

**Keywords:** AMPK, ferroptosis, non‐small cell lung cancer, TRIM11

## Abstract

Lung cancer is one of the leading causes of cancer‐related deaths worldwide, with non‐small cell lung cancer (NSCLC) being the most prevalent type. This study investigates the role of TRIM11 gene in NSCLC and its underlying mechanism. NSCLC patients were recruited from our hospital and showed upregulated TRIM11 mRNA and protein expressions. Patients with high TRIM11 expression had lower survival rates. TRIM11 gene was found to promote cell proliferation and reduce ROS‐induced ferroptosis in NSCLC. Additionally, TRIM11 gene induced AMPK expression and its regulation affected TRIM11's effects on cell proliferation and ferroptosis in NSCLC. IP analysis revealed that TRIM11 protein interacted with AMPK protein in NSCLC. These data confirmed that TRIM11 promotes cell proliferation and reduces ROS‐induced ferroptosis in NSCLC through AMPK. Hence, TRIM11 is a potential target for the treatment of NSCLC and other cancers.

## Graphical abstract

TRIM11 promoted cell proliferation and reduced ROS‐induced ferroptosis of NSCLC by AMPK. This infers that TRIM11 is potential targets to be used in the treatment of NSCLC or other cancers.

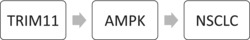

## 1 INTRODUCTION

Lung cancer is the second most common cancer in terms of incidence and the leading cause of cancer‐related deaths worldwide.^1^ According to the 2020 GLOBOCAN report on global cancer statistics, there were 2.207 million new cases of lung cancer worldwide, ranking second only to breast cancer.^2^ The number of deaths due to lung cancer was 1.796 million, making it the most deadly cancer type.^3^ In China, lung cancer is not only the most common cancer in terms of incidence but also mortality.^3^ In 2020, the estimated number of new cases of lung cancer in China was 816 000, accounting for 17.9% of all new cancer cases, and the number of deaths was 715 000, accounting for 23.8% of all cancer deaths.^4^ Based on age‐standardized rates (ASR), the incidence rate of lung cancer in Chinese men and women was 47.8 per 100 000 and 22.8 per 100 000, respectively, and the mortality rate was 41.8 per 100 000 and 19.7 per 100 000, respectively.^5^

The occurrence of cancer is related to many factors, such as smoking and passive smoking, silica, asbestos, tin, coal tar, diet, oil fume, air pollution, etc.^6^ Both smoking and passive smoking exposure are important risk factors for lung cancer, and smoking can increase the risk of lung cancer by more than three times.^7^ In our country, 72.4% of non‐smokers are exposed to passive smoking, which is extensive, and the impact on non‐smokers' lung cancer cannot be ignored.^6^ Lung cancer ranks first in the death and incidence of malignant tumors in China.^8^ Lung cancer is closely related to smoking and passive smoking exposure.^6^ In recent years, China has taken a series of measures to control smoking and prevent lung cancer, but the burden of lung cancer is still serious, the smoking rate of men is still high, and non‐smokers are very widely exposed to passive smoking.^9^

Ferroptosis is a recently discovered programmed cell death mode that is distinct from apoptosis, necrosis, and autophagy.^10^ Its primary mechanism involves the catalysis of lipid peroxidation of highly expressed unsaturated fatty acids in cells under the influence of divalent iron or lipoxygenase, leading to cell death.^11^ Ferroptosis is implicated in the development of many diseases, including tumors, neurodegenerative diseases, rheumatoid arthritis, ischemia–reperfusion, and cardiovascular‐related diseases.^11^ Lung cancer is the most deadly malignant tumor globally, and its treatment strategy is continuously evolving.^11^ In recent years, an increasing number of research findings indicate a close connection between ferroptosis and non‐small cell lung cancer (NSCLC).^12^ Some molecules that play a significant regulatory role in the development, treatment, and onset of NSCLC, such as KRAS, TP53, and EGFR, are also implicated in ferroptosis.^12^ Additionally, ferroptosis in NSCLC is closely tied to chemotherapy, radiotherapy, and immunotherapy. Some preclinical studies have shown that ferroptosis can serve as a “catalyst,” and combining ferroptosis with the aforementioned treatment methods can significantly enhance treatment effectiveness, opening up a new research avenue for NSCLC treatment.^13^

AMPK is an energy sensor in the body that enhances energy utilization, regulates autophagy and mitochondrial fusion and fission, and participates in various pathophysiological activities.^14^ Recent studies have demonstrated that AMPK activation can alleviate brain I/R injury by regulating energy metabolism, oxidative stress, and mitochondrial function.^15^, ^16^ Furthermore, the use of AMPK activators in tumor cells has been reported to regulate lipid synthesis and metabolism, thus reducing the incidence of cell ferroptosis.^17^ Additionally, it has been reported that upregulating the expression of p‐AMPK can reduce lipid peroxide levels after I/R and subsequent nerve injury in rats.^18^

The TRIM11 protein, a member of the TRIM family, is involved in regulating cell apoptosis, proliferation, and cell cycle.^19^ As a tumor promoting factor, TRIM11 can enhance tumor cell proliferation and invasion through various mechanisms.^20^ High expression of TRIM11 was observed in advanced stage gliomas, while low expression was associated with poor differentiation and favorable prognosis.^21^ Additionally, TRIM11 can activate MAPK or PI3K/AKT signaling pathways to promote tumor cell proliferation, invasion, and migration.^22^ Shang et al. identified that TRIM11 suppresses ferritinophagy in pancreatic ductal adenocarcinoma.^23^ So, this study elucidates the role and potential mechanism of TRIM11 in ferroptosis of NSCLC.

## 2 MATERIALS AND METHODS

### 2.1 Patients with NSCLC

This study was approved by the ethics committee of our hospital. All the serum samples (number = 40) were immediately snap frozen in liquid nitrogen and stored at −80°C. Pathological evaluation was performed according to the WHO classification by two experienced clinical pathologists. All methods were carried out in accordance with relevant guidelines and regulations. Informed consent was obtained from all participants.

### 2.2 Cell culture and transfection

BEAS‐2B, NCI‐H1437, NCI‐H1299, NCI‐H322, and A549 cells were performed in compliance with ATCC protocols and incubated in a 5% CO_2_ atmosphere at 37°C. Plasmids were transfected into NSCLC cell lines using Lipofectamine 2000.

### 2.3 Microarray experiments

Microarray experiments were performed at the Genminix Informatics (China). Gene expression profiles were analyzed with the Human Exon 1.0 ST GeneChip (Affymetrix).

### 2.4 Quantitative polymerase chain reaction (qPCR)

Total RNAs were isolated with RNA isolator total RNA extraction reagent (Takara), and cDNA was synthesized using PrimeScipt RT Master Mix (Takara). qPCR w**as** performed with the ABI Prism 7500 sequence detection system according to the Prime‐ScriptTM RT detection kit. Relative levels of the sample mRNA expression were calculated and expressed as 2‐ΔΔCT. Primer sequence: TRIM11: forward: 5**′**‐GTGCCTATGGAGCTGAGGAC‐3′; reverse: 5**′**‐CAGGATCAGCTCAGGGTTG‐3′; β‐actin: forward: 5**′**‐TACCTCATGAAGATCCTCACC‐3′; reverse: 5**′**‐TTTCGTGGATGCCACAGGAC‐3′. High TRIM11 expression of NSCLC patients ≥3 fold of TRIM11 expression of normal patients, and low TRIM11 expression of NSCLC patients <3**‐**fold of TRIM11 expression of normal patients.

### 2.5 Immunofluorescent staining

Cells were fixed with 4% paraformaldehyde for 15 min and incubated with using 0.15% Triton X‐100 for 15 min at room temperature. Cells were incubated with TRIM11 (ab191217, 1:500, abcam) and p‐AMPK (3934, 1:500, Cell Signaling Technology, Inc.) at 4°C overnight after blocking with 5% BSA for 1 h. Cells was incubated with goat anti‐rabbit IgG‐cFL 488 or anti‐rabbit IgG‐cFL 555 antibody (1:100) for 2 h at room temperature and stained with DAPI for 15 min and washed with PBS for 15 min. The images of cells were obtained using a Zeiss Axioplan 2 fluorescent microscope (carl Zeiss AG, Oberkochen, Germany).

### 2.6 Proliferation assay and EDU staining

For cell counting kit‐8 (CCK‐8), after 48 h of transfection, a total of approximately 2 × 10^3^ cells/well was seeded in 96‐well plate. After culturing at indicated time (0, 1, 2, 3, and 4 day), the cellular proliferation was detected using CellTiter‐GloR Luminescent Cell Viability Assay (Promega, Madison, WI, USA) according to manufacturer's instructions.

For ethynyl deoxyuridine (EdU) incorporation assay, EdU (10 mM) was added to each well, and cells were fixed with 4% formaldehyde for 30 min. After washing, EdU was detected with Click‐iTR EdU Kit, and images were visualized using fluorescent microscope (Olympus).

### 2.7 Western blot

Tissue or cells samples were lysed with ice‐cold RIPA buffer with complete protease and phosphatase inhibitors. The protein concentrations were measured using BCA protein assay kit. Total proteins were separated by SDS–PAGE and transferred onto polyvinylidene difluoride (PVDF) membranes. The membranes were incubated with primary antibodies: AMPK (1:1000, ab169197, abcam) and p‐AMPK (1:1000, 3934, Cell Signaling Technology, Inc.), TRIM11 (1:1000, ab191217, abcam), GPX4 (1:1000, ab267373, abcam), and β‐Actin (1:5000, Santa Cruz Biotechnology) after blocking with 5% BSA in TBS, followed by incubation with peroxidase‐conjugated secondary antibodies (Santa Cruz Biotechnology). The signals were detected with the ECL system and exposed by the ChemiDoc XRS system with Image Labsoftware (Bio‐rad).

### 2.8 Statistical analyses

Survival analysis was performed using the Kaplan–Meier method and log‐rank test for survival curves. GraphPad Prism 6 was used for the statistical analysis. *p* < 0.05 was considered statistically significant. Comparisons of data between groups were followed using Student's *t* test or one‐way analysis of variance (ANOVA), followed by Tukey's post hoc test.

## 3 RESULTS

### 3.1 TRIM11 expression level in patients with NSCLC

**FIGURE 1:**
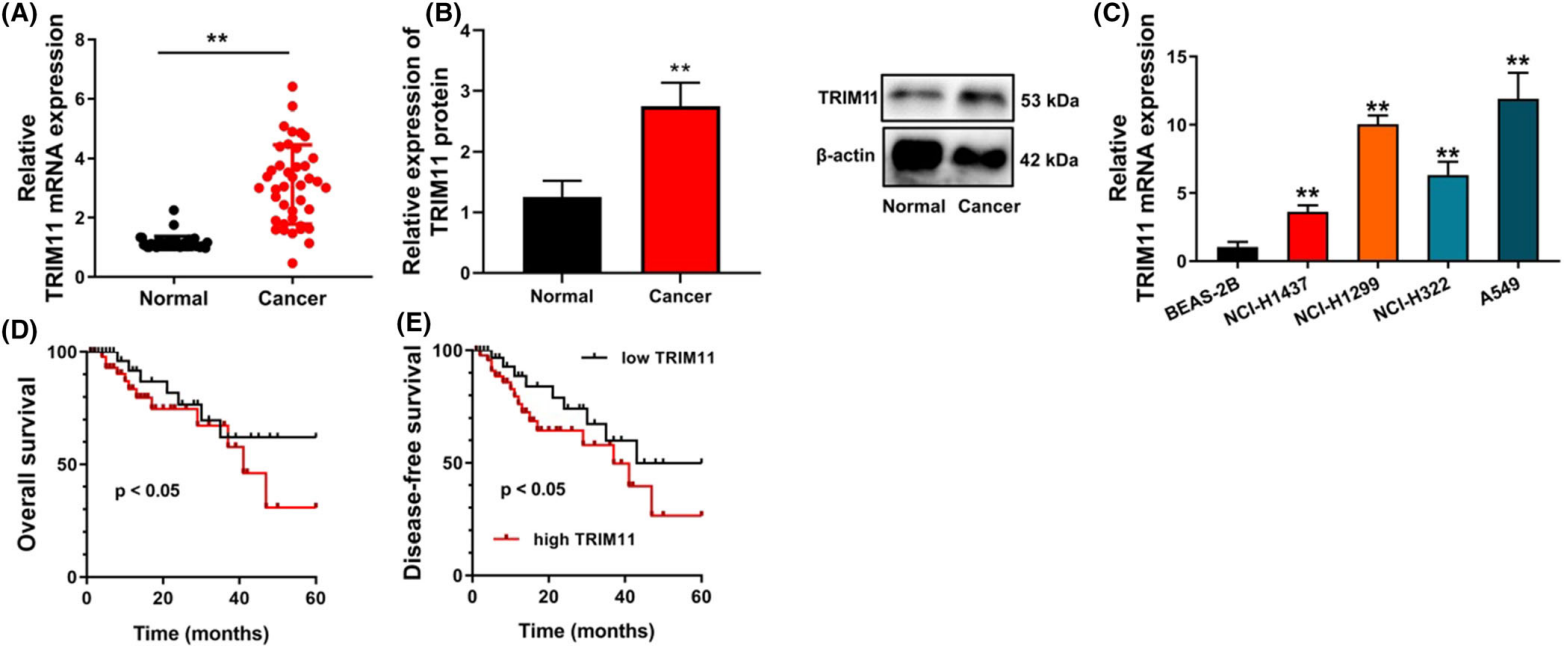
TRIM11 expression level in patients with NSCLC. TRIM11 mRNA and protein expressions in patients with NSCLC (A and B), TRIM11 mRNA expressions in NSCLC cell line (C), and OS and DFS (D and E). **p < 0.01 compared with normal volunteers group or BEAS‐2B group.

We first evaluated the expression of TRIM11 in patients with NSCLC. The results showed that TRIM11 mRNA and protein expressions were upregulated in patients with NSCLC, compared with normal (Figure 1A,B). Furthermore, TRIM11 mRNA expressions in NSCLC cell lines were higher compared to normal lung cells (Figure 1C). Kaplan–Meier survival analysis revealed that NSCLC patients with high TRIM11 expression had a lower survival rate (Figure 1E,F). These findings suggest that TRIM11 plays an activating role in NSCLC.

### 3.2 TRIM11 gene promoted cell proliferation of NSCLC

**FIGURE 2:**
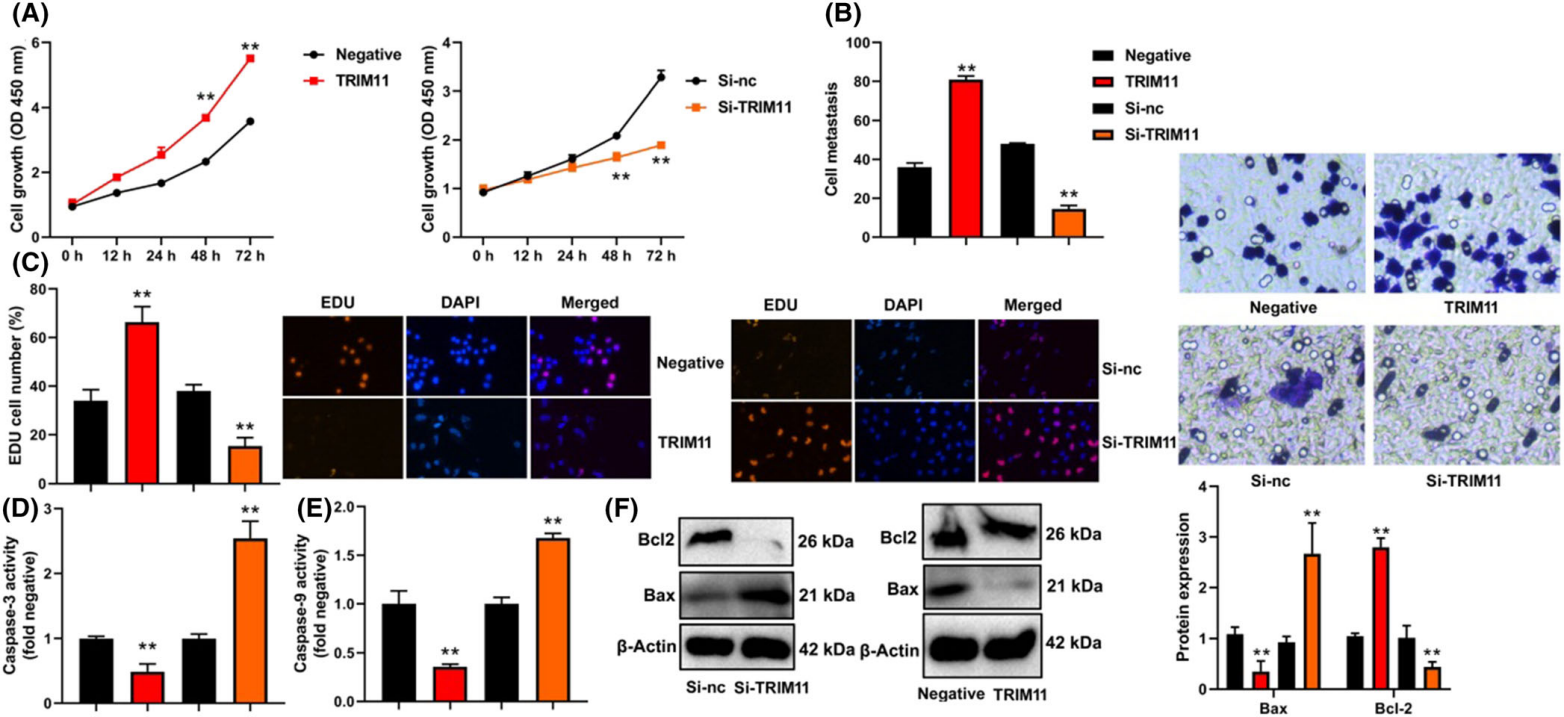
TRIM11 gene promoted cell proliferation of NSCLC. Cell growth (A), migration rate (B), EDU assay (C), and caspase‐3/9 activity level (D and E). **p < 0.01 compared with negative group or si‐nc group.

We conducted experiments to determine the effect of TRIM11 on cell proliferation in NSCLC. The results showed that TRIM11 gene facilitated cell proliferation, as evidenced by increased EDU cells and cell metastasis in NSCLC, compared with negative (Figure 2A–C). On the other hand, si‐TRIM11 inhibited cell proliferation, as demonstrated by reduced EDU cells and cell metastasis in NSCLC, compared with si‐nc (Figure 2A–C). Furthermore, TRIM11 gene upregulation decreased the activity levels of caspase‐3/9 and Bax protein expression and induced Bcl‐2 protein expression in NSCLC, compared with negative (Figure 2D–F), while si‐TRIM11 promoted caspase‐3/9 activity levels and Bax protein expression and suppressed Bcl‐2 protein expression in NSCLC, compared with si‐nc (Figure 2D–F).

### 3.3 TRIM11 gene reduced ROS‐induced ferroptosis of NSCLC

**FIGURE 3:**
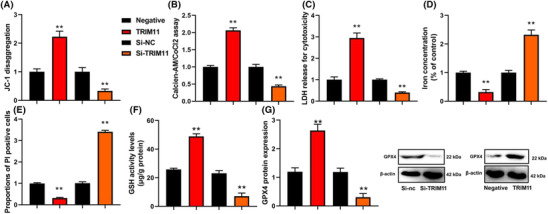
TRIM11 gene reduced ROS‐induced ferroptosis of NSCLC. JC‐1 disaggregation (A), calcein‐AM/CoCl2 levels (B), LDH activity (C), iron concentration levels (D), calcein/PI (E), GSH (F), and GPX4 protein expression (G). **p < 0.01 compared with negative group or si‐nc group.

We then investigated the impact of TRIM11 on the ferroptosis induced by ROS in NSCLC. We found that upregulation of TRIM11 gene activity increased mitochondrial damage as evidenced by JC‐1 disaggregation and increased MPT as observed in the calcein AM/CoCl2 assay. Additionally, we observed a reduction in LDH activity levels and a decrease in the proportion of PI‐positive cells. Furthermore, TRIM11 gene upregulation inhibited iron concentration levels and expanded GSH and GPX4 protein expressions in NSCLC, compared with negative (Figure 3). On the other hand, silencing of the TRIM11 gene reduced mitochondrial damage and MPT, increased LDH activity levels and the proportion of PI‐positive cells, promoted iron concentration levels, and suppressed GSH and GPX4 protein expressions in NSCLC, compared with negative (Figure 3). Overall, our findings suggest that TRIM11 gene activity reduces ROS‐induced ferroptosis in NSCLC by inhibiting mitochondrial damage.

### 3.4 TRIM11 gene induced AMPK expression in NSCLC

**FIGURE 4:**
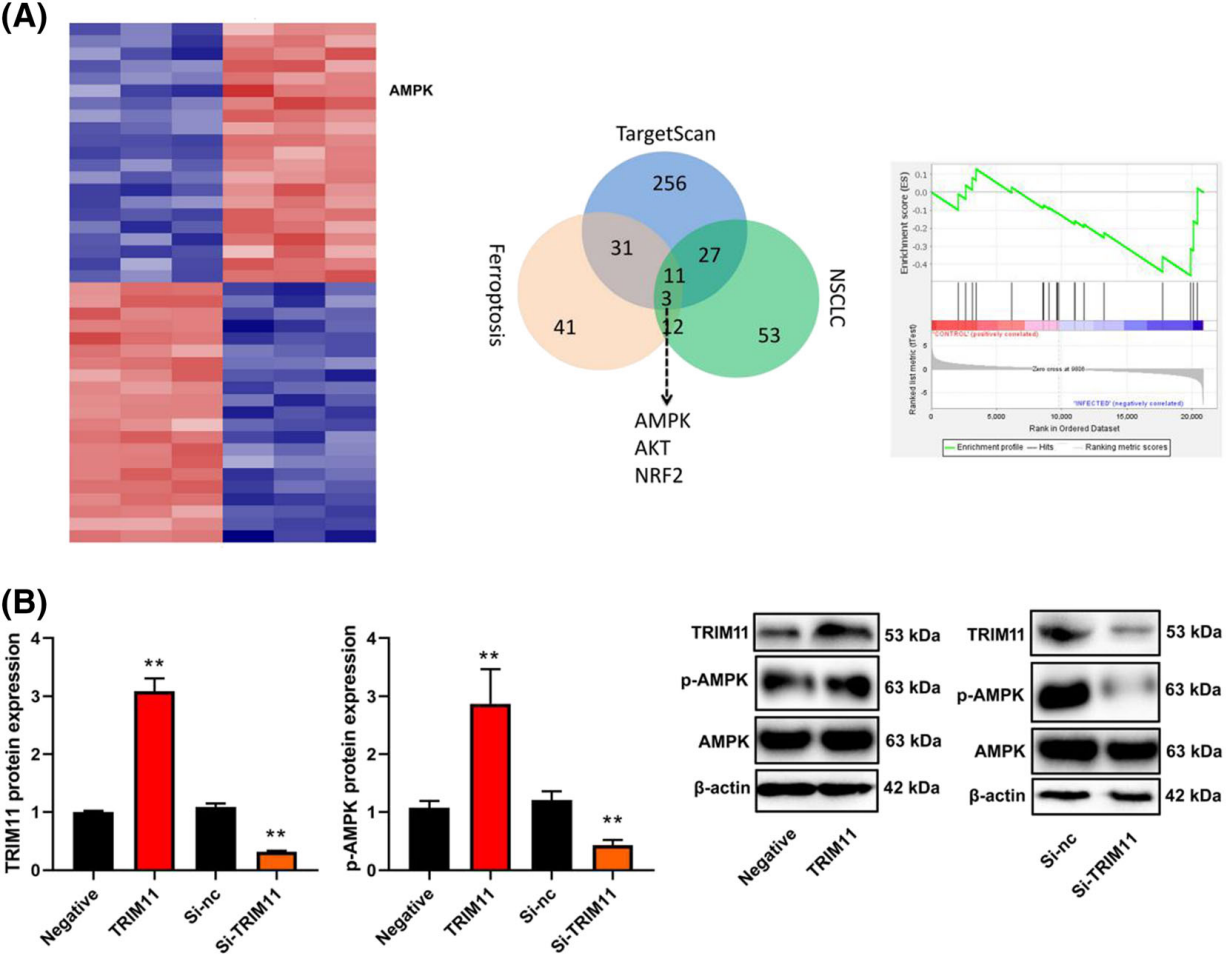
TRIM11 gene induced AMPK expression in NSCLC. Results of microarray analysis and KEGG terms (A) and TRIM11 and p‐AMPK protein expression (B). **p < 0.01 compared with negative group or si‐nc group.

The study delved into the mechanism behind TRIM11's role in ferroptosis in NSCLC through microarray experiments. The results indicated that AMPK could potentially be a target of TRIM11 in ferroptosis of NSCLC (as depicted in Figure 4A). Upregulation of TRIM11 led to an increase in p‐AMPK protein expression, whereas si‐TRIM11 decreased p‐AMPK protein expression in NSCLC (as seen in Figure 4B). These findings shed light on the potential involvement of AMPK in TRIM11‐mediated ferroptosis in NSCLC.

### 3.5 The regulation of AMPK affected the effects of TRIM11 on cell proliferation of NSCLC

**FIGURE 5:**
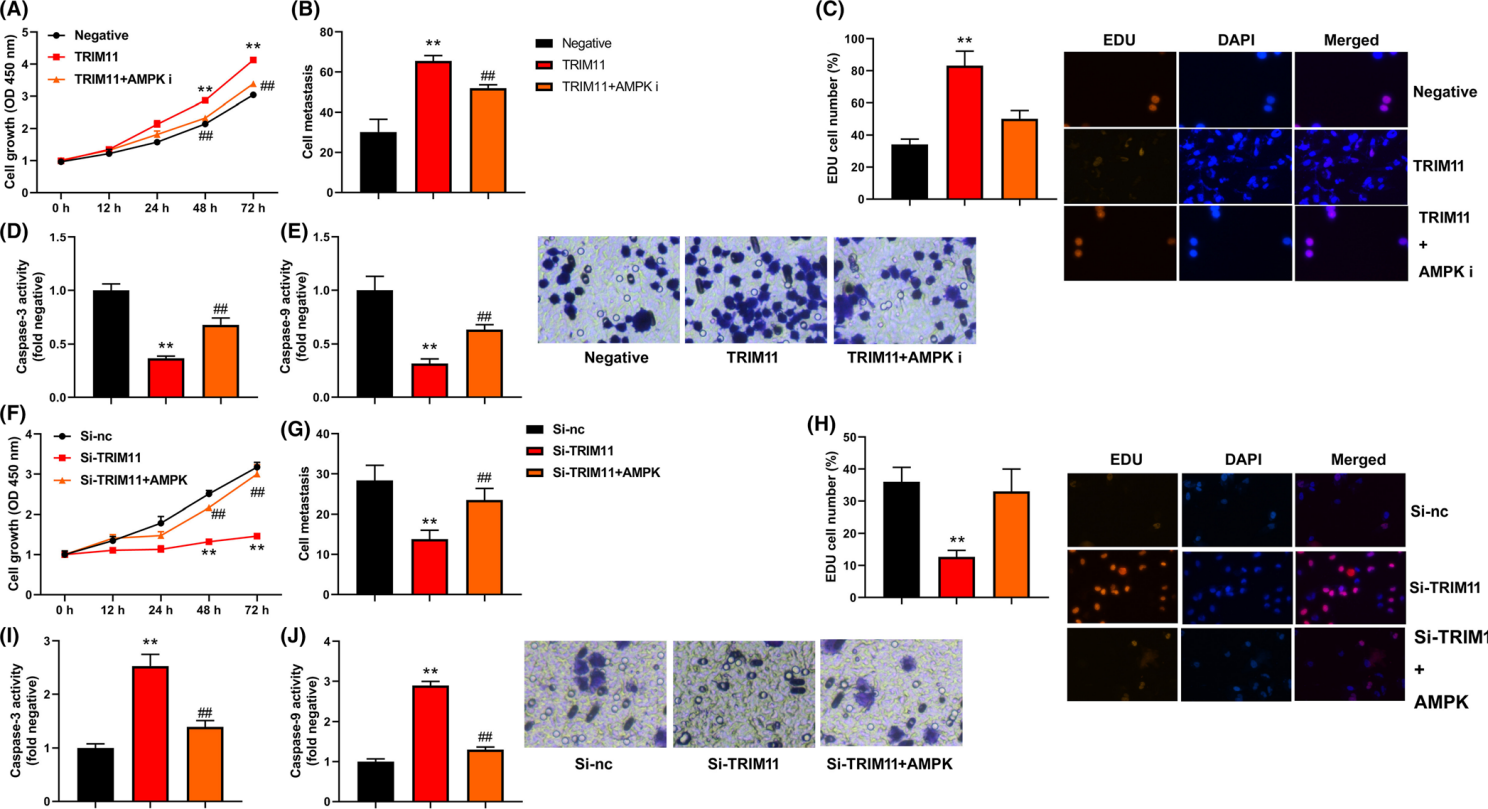
The regulation of AMPK affected the effects of TRIM11 on cell proliferation of NSCLC. Cell growth (A), migration rate (B), EDU assay (C), and caspase‐3/9 activity level (D and E) in vitro model by TRIM11 + AMPK inhibitor; cell growth (F), migration rate (G), EDU assay (H), and caspase‐3/9 activity level (I and J) in vitro model by si‐TRIM11 + AMPK agonist. **p < 0.01 compared with negative group or si‐nc group; ^##^ *p* < 0.01 compared with TRIM11 or si‐TRIM11 group.

The study investigated the potential mechanism by which TRIM11 affects the progression of gastric NSCLC through AMPK expression. The use of an AMPK inhibitor (100 nM of AMPK‐IN‐3) resulted in reduced cell proliferation and EDU cells, inhibited cell metastasis, and increased caspase‐3/9 activity levels in NSCLC with upregulated TRIM11 gene expression (as shown in Figure 5A–E). On the other hand, an AMPK agonist (20 nM of Ampkinone) reduced cell proliferation and EDU cells, increased cell metastasis, and decreased caspase‐3/9 activity levels in NSCLC with downregulated TRIM11 gene expression (as depicted in Figure 5F–J). These findings suggest a potential role of AMPK in the regulation of TRIM11‐mediated cell progression in gastric NSCLC.

### 3.6 The regulation of AMPK affected the effects of TRIM11 on ferroptosis of NSCLC

**FIGURE 6:**
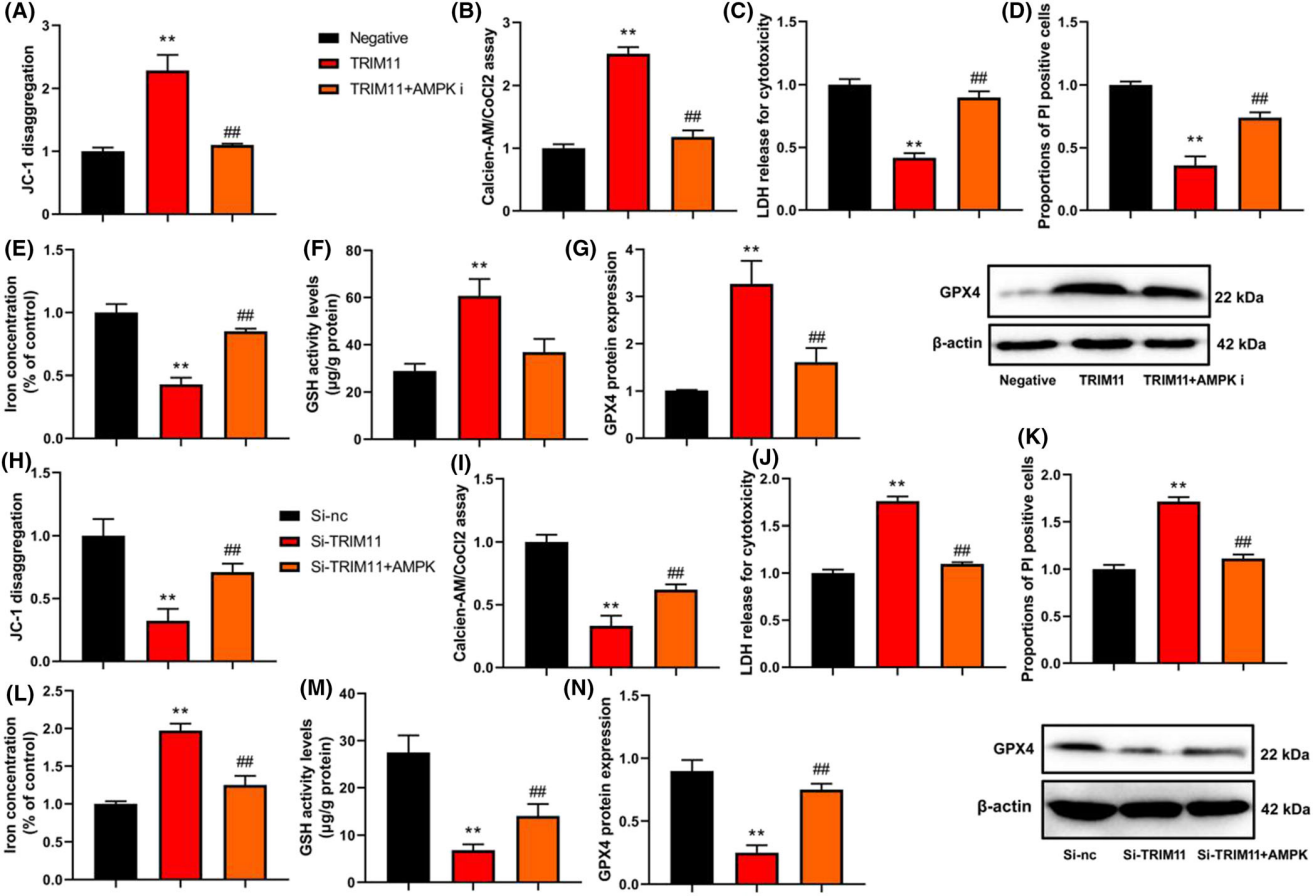
The regulation of AMPK affected the effects of TRIM11 on ferroptosis of NSCLC. JC‐1 disaggregation (A), calcein‐AM/CoCl2 levels (B), LDH activity (C), and iron concentration levels (D), calcein/PI (E), GSH (F), and GPX4 protein expression (G) in vitro model by TRIM11 + AMPK inhibitor; JC‐1 disaggregation (H), calcein‐AM/CoCl2 levels (I), LDH activity (J), and iron concentration levels (K), calcein/PI (L), GSH (M), and GPX4 protein expression (N) in vitro model by si‐TRIM11 + AMPK agonist. **p < 0.01 compared with negative group or si‐nc group; ^##^ *p* < 0.01 compared with TRIM11 or si‐TRIM11 group.

In addition, we observed that the AMPK inhibitor reduced mitochondrial damage and mitochondrial permeability transition (MPT), increased lactate dehydrogenase (LDH) activity levels and the proportion of propidium iodide (PI)‐positive cells, promoted iron concentration levels, and suppressed glutathione (GSH) and glutathione peroxidase 4 (GPX4) protein expressions in NSCLC with upregulated TRIM11 gene expression (as shown in Figure 6A–G). Conversely, the AMPK agonist increased mitochondrial damage and MPT, reduced LDH activity levels and the proportion of PI‐positive cells, inhibited iron concentration levels, and expanded GSH and GPX4 protein expressions in NSCLC with downregulated TRIM11 gene expression (as depicted in Figure 6H–N). These results suggest that AMPK may play a crucial role in the regulation of TRIM11‐mediated cell progression in gastric NSCLC by modulating cellular metabolism and redox homeostasis.

### 3.7 TRIM11 protein interlinked AMPK protein

**FIGURE 7:**
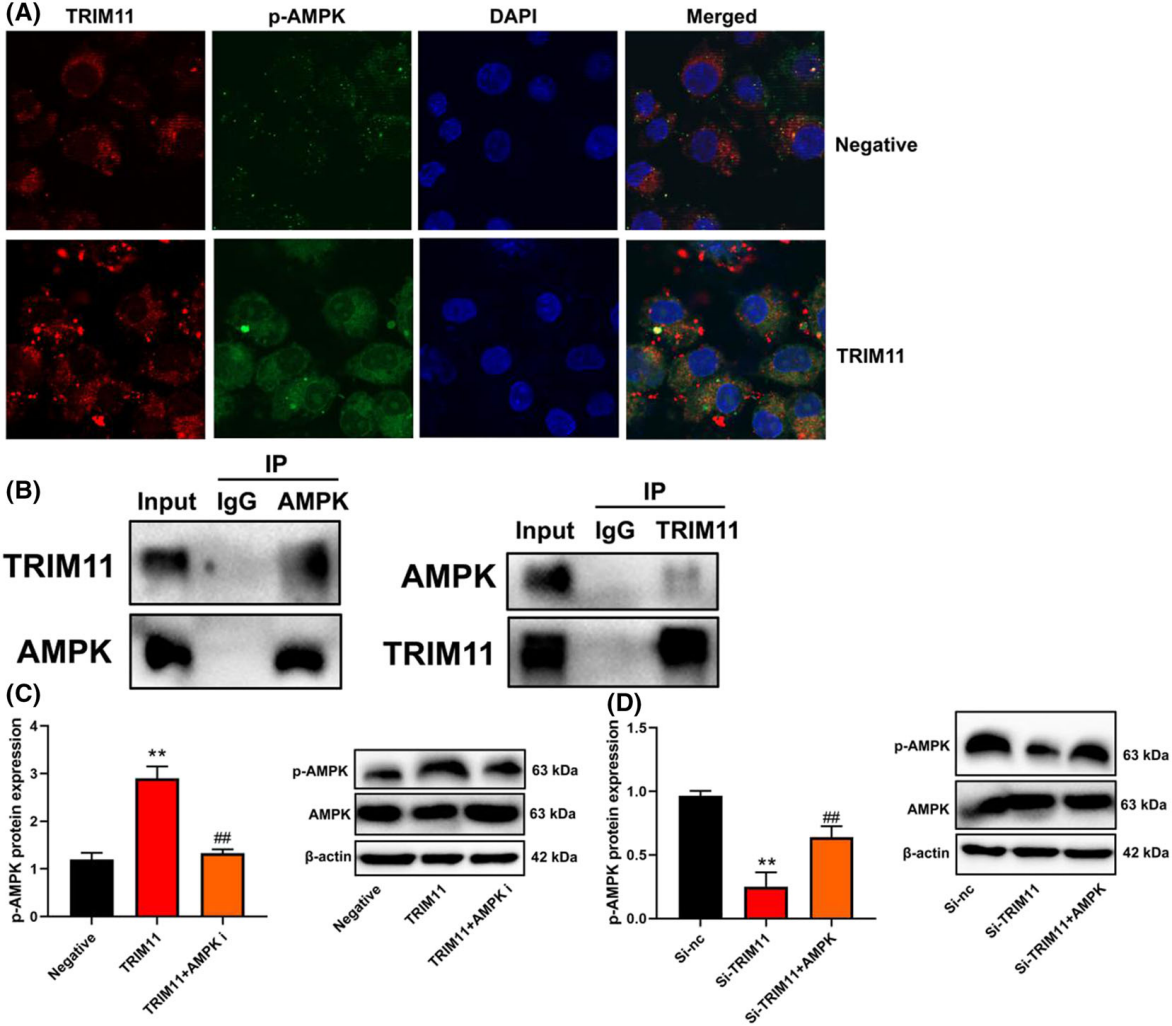
TRIM11 protein interlinked AMPK protein. TRIM11 and p‐AMPK expression (confocal microscope, A), TRIM11 protein interlinked AMPK protein (IP, B), p‐AMPK expression (C and D). **p < 0.01 compared with negative group or si‐nc group; ^##^ *p* < 0.01 compared with TRIM11 or si‐TRIM11 group.

We investigated whether ferroptosis is functionally involved in TRIM11/AMPK‐mediated treatment for NSCLC. Confocal microscopy revealed that TRIM11 upregulation increased p‐AMPK expression in NSCLC (as shown in Figure 7A). Immunoprecipitation (IP) analysis demonstrated that TRIM11 protein interacted with AMPK protein in NSCLC (as depicted in Figure 7B). Furthermore, the AMPK inhibitor suppressed p‐AMPK expression in NSCLC (as demonstrated in Figure 7C), while the AMPK agonist induced p‐AMPK expression in NSCLC (as illustrated in Figure 7D). These findings suggest that TRIM11 promotes cell proliferation in NSCLC by inhibiting ferroptosis through AMPK expression.

## 4 DISCUSSION

Lung cancer is a prevalent and deadly malignancy worldwide, with non‐small cell lung cancer (NSCLC) is the most common histological type of lung cancer, accounting for 85%.^24^, ^25^ In China, lung cancer is the most common cancer and the leading cause of cancer‐related deaths.^24^, ^25^ Unfortunately, NSCLC is often diagnosed in advanced stages, leading to poor prognosis.^26^ TRIM11, which has been linked to the migration and invasion of nasopharyngeal carcinoma, was found to be upregulated in NSCLC patients at both the mRNA and protein levels. Patients with high TRIM11 expression had lower survival rates.^27^ Therefore, inhibiting TRIM11 may offer protection against NSCLC. Our study demonstrated that TRIM11 mRNA and protein expressions were upregulated in NSCLC patients. Furthermore, patients with high TRIM11 expression had lower survival rates. Previous research by Zhao et al. also showed that TRIM11 facilitated the migration and invasion of nasopharyngeal carcinoma cells.^28^ These results suggest that TRIM11 is upregulated in NSCLC and that inhibiting TRIM11 may offer protection against this disease. In this study, we only analyzed the serum expression of TRIM11, which was one insufficient for this experiment. We will analyze TRIM11 expression in tissue of patients with NSCLC.

Lung cancer is a prevalent and deadly malignancy worldwide, with non‐small cell lung cancer (NSCLC) accounting for about 85% of all newly diagnosed cases, making it the main pathological type of lung cancer.^29^ Small cell lung cancer is the other main type. In China, lung cancer is the most common cancer and the leading cause of cancer‐related deaths. Unfortunately, NSCLC is often diagnosed in advanced stages, leading to poor prognosis.^30^ In 2012, we first discovered and proposed “ferroptosis”—a cell death mode different from apoptosis, necrosis, and autophagy, which is characterized by abnormal lipid peroxidation and accumulation of reactive oxygen species (ROS).^31^ In ferroptosis cells, mitochondria usually show a decrease in volume, an increase in membrane density, and a reduction or disappearance of cristae. Although ferroptosis may play a dual regulatory role in promoting or inhibiting the occurrence of tumors, small molecular drugs induced ferroptosis may be a potential anti‐tumor treatment strategy.^32^ Ferroptosis is a newly discovered iron dependent programmed death.^33^ More and more studies have shown that ferroptosis plays an important role in the progress of tumors, especially NSCLC, and targeting ferroptosis is a potential therapeutic strategy for lung cancer.^33^ To provide new research directions for the treatment of NSCLC, it is important to understand the mechanism and regulatory network of ferroptosis, the role of NSCLC‐related regulatory genes (such as KRAS, TP53, EGFR, etc.) in ferroptosis, and the relationship between ferroptosis and chemotherapy, radiotherapy, and immunotherapy. We have revealed that the TRIM11 gene promotes cell proliferation and reduces ROS‐induced ferroptosis of NSCLC. Shang et al have identified that TRIM11 suppresses ferritinophagy of pancreatic ductal adenocarcinoma.^28^

GPX4 is produced on the cell membrane and can directly reduce the level of phospholipid peroxide.^34^ When the activity of GPX4 decreases, it cannot inhibit the production of ROS in the cell, resulting in the accumulation of lipid peroxide and the production of lethal ROS, thus inducing cell ferroptosis.^35^ Cystine can be oxidized to cysteine after entering the cell, which can then be combined with glycine to generate glutathione (GSH) under the action of glutamate‐cysteine ligase and glutathione synthetase. There are two forms of glutathione: oxidized and reduced,^36^ which can participate in the regulation of intracellular oxidation balance.^37^ Glutathione reductase can catalyze their mutual transformation. This study revealed that the regulation of AMPK affected the effects of TRIM11 on ferroptosis of NSCLC. Therefore, TRIM11 might contribute to the inhibition of ferroptosis in NSCLC.

AMPK is an important energy regulator. Under low energy conditions, AMPK phosphorylates specific enzymes and growth control nodes to enhance the efficiency of ATP utilization and maintain the energy supply of the body.^38^ AMPK plays a crucial role in cell physiology and the pathological development of chronic diseases, including cancer.^39^ AMPK phosphorylation can inhibit the expression of SCD1, reduce lipid peroxidation, and inhibit the occurrence of ferroptosis.^40^ Ferroptotic cells will produce ROS, and ROS production may also lead to activation of AMPK phosphorylation, and negative feedback can inhibit cell ferroptosis.^41^ This study showed that TRIM11 protein interlinks with AMPK protein, and TRIM11 gene induces AMPK expression in NSCLC. Liu et al. showed that TRIM11 binds to and ubiquitinates AMPK subunit.^42^ Therefore, our results revealed that the inhibition of TRIM11 protects NSCLC via the inhibition of AMPK.

In conclusion, our study demonstrated that TRIM11 promotes cell proliferation and reduces ROS‐induced ferroptosis in NSCLC by activating AMPK. These findings provide a new understanding of the role of TRIM11 in NSCLC and suggest a novel target for NSCLC treatment. Inhibition of TRIM11 may be a potential strategy for the treatment of early‐stage NSCLC and other cancers. Further studies are needed to explore the therapeutic potential of targeting TRIM11 in cancer treatment.

## AUTHOR CONTRIBUTIONS

Zheng Liang designed the experiments. Jian Li and Guoliang Zhang performed the experiments. Jian Li and Guoliang Zhang collected and analyzed the data. Zheng Liang and Menghui Chen drafted manuscript. All authors read and approved the final manuscript.

## CONFLICT OF INTEREST STATEMENT

The authors state that there are no financial, personal, or professional conflicts of interests that may hinder this work.

## ETHICS STATEMENT

All patients were informed and signed informed consent voluntarily. This study was approved by the Ethics Committee of The Third Hospital of Shijiazhuang and complied with the guidelines outlined in the declaration of Helsinki were followed. The written consent was received from all participants.

## Data Availability

The data that support the findings of this study are available from the corresponding author upon reasonable request.

## References

[1] Rosner S , Reuss JE , Zahurak M , et al. Five‐year clinical outcomes after neoadjuvant nivolumab in resectable non‐small cell lung cancer. Clin Cancer Res. 2023;29(4):705‐710. doi:10.1158/1078-0432.CCR-22-2994 36794455 PMC9932577

[2] Sharman Moser S , Tanser F , Siegelmann‐Danieli N , Apter L , Chodick G , Solomon J . The reimbursement process in three national healthcare systems: variation in time to reimbursement of pembrolizumab for metastatic non‐small cell lung cancer. J Pharm Policy Pract. 2023;16(1):22. doi:10.1186/s40545-023-00529-0 36797806 PMC9936745

[3] Wespiser M , Marguier A , Lecoester B , et al. Response to chemoimmunotherapy is associated with expansion of systemic antitumor CD4+ Th1 response in metastatic non‐small cell lung cancer. J Immunother. 2023;46(7):279‐283. doi:10.1097/CJI.0000000000000454 36799899

[4] Vaseghi G , Rashidi N , Zare N , et al. Effects of methadone on the toll‐like receptor 4 expression in human non‐small cell lung carcinoma A549 cell line using in‐silico and in vitro techniques. Adv Biomed Res. 2022;11(1):122. doi:10.4103/abr.abr_97_21 36798925 PMC9926039

[5] Socinski MA , Jotte RM , Cappuzzo F , et al. Association of immune‐related adverse events with efficacy of atezolizumab in patients with non‐small cell lung cancer: pooled analyses of the phase 3 IMpower130, IMpower132, and IMpower150 randomized clinical trials. JAMA Oncol. 2023;9(4):527‐535. doi:10.1001/jamaoncol.2022.7711 36795388 PMC9936386

[6] Liu J , Chen SJ , Hsu SW , et al. MARCKS cooperates with NKAP to activate NF‐kB signaling in smoke‐related lung cancer. Theranostics. 2021;11(9):4122‐4136. doi:10.7150/thno.53558 33754052 PMC7977464

[7] de Groot P , Munden RF . Lung cancer epidemiology, risk factors, and prevention. Radiol Clin North Am. 2012;50(5):863‐876. doi:10.1016/j.rcl.2012.06.006 22974775

[8] Middleton G , Fletcher P , Popat S , et al. The National Lung Matrix Trial of personalized therapy in lung cancer. Nature. 2020;583(7818):807‐812. doi:10.1038/s41586-020-2481-8 32669708 PMC7116732

[9] Corrales L , Rosell R , Cardona AF , Martín C , Zatarain‐Barrón ZL , Arrieta O . Lung cancer in never smokers: the role of different risk factors other than tobacco smoking. Crit Rev Oncol Hematol. 2020;148:102895. doi:10.1016/j.critrevonc.2020.102895 32062313

[10] Santarpia M , Aguilar A , Chaib I , et al. Non‐small‐cell lung cancer signaling pathways, metabolism, and PD‐1/PD‐L1 antibodies. Cancers (Basel). 2020;12(6):1475. doi:10.3390/cancers12061475 32516941 PMC7352732

[11] Han B , Liu Y , Zhang Q , Liang L . Propofol decreases cisplatin resistance of non‐small cell lung cancer by inducing GPX4‐mediated ferroptosis through the miR‐744‐5p/miR‐615‐3p axis. J Proteomics. 2023;274:104777. doi:10.1016/j.jprot.2022.104777 36427803

[12] Tang X , Ding H , Liang M , et al. Curcumin induces ferroptosis in non‐small‐cell lung cancer via activating autophagy. Thorac Cancer. 2021;12(8):1219‐1230. doi:10.1111/1759-7714.13904 33656766 PMC8046146

[13] Zou J , Wang L , Tang H , Liu X , Peng F , Peng C . Ferroptosis in non‐small cell lung cancer: progression and therapeutic potential on it. Int J Mol Sci. 2021;22(24):13335. doi:10.3390/ijms222413335 34948133 PMC8704137

[14] Dong S , Zheng L , Jiang T . Loss of lactate/proton monocarboxylate transporter 4 induces ferroptosis via the AMPK/ACC pathway and inhibition of autophagy on human bladder cancer 5637 cell line. J Oncol. 2023;2023:2830306. doi:10.1155/2023/2830306 36718218 PMC9884169

[15] Jin C , Tan K , Yao Z , et al. A novel anti‐osteoporosis mechanism of VK2: interfering with ferroptosis via AMPK/SIRT1 pathway in type 2 diabetic osteoporosis. J Agric Food Chem. 2023;71(6):2745‐2761. doi:10.1021/acs.jafc.2c05632 36719855

[16] Li D , Zhang G , Wang Z , et al. Idebenone attenuates ferroptosis by inhibiting excessive autophagy via the ROS‐AMPK‐mTOR pathway to preserve cardiac function after myocardial infarction. Eur J Pharmacol. 2023;943:175569. doi:10.1016/j.ejphar.2023.175569 36740037

[17] Yi X , Long X , Liu C . Activating autophagy and ferroptosis of 3‐chloropropane‐1,2‐diol induces injury of human umbilical vein endothelial cells via AMPK/mTOR/ULK1. Mol Med Rep. 2023;27(3):76. doi:10.3892/mmr.2023.12963 36799162 PMC9950850

[18] Lu Q , Yang L , Xiao JJ , et al. Empagliflozin attenuates the renal tubular ferroptosis in diabetic kidney disease through AMPK/NRF2 pathway. Free Radic Biol Med. 2023;195:89‐102. doi:10.1016/j.freeradbiomed.2022.12.088 36581059

[19] Hanna J , Ko JS , Billings SD , et al. Cutaneous melanocytic tumor with CRTC1::TRIM11 translocation: an emerging entity analyzed in a series of 41 cases. Am J Surg Pathol. 2022;46(11):1457‐1466. doi:10.1097/PAS.0000000000001952 35993578

[20] Yang L , Yin Z , Wei J , et al. Cutaneous melanocytic tumour with CRTC1::TRIM11 fusion in a case with recurrent local lymph node and distant pulmonary metastases at early stage: aggressive rather than indolent? Histopathology. 2023;82(2):368‐371. doi:10.1111/his.14812 36177516

[21] Vest BE , Harview CL , Liu V , et al. Cutaneous melanocytic tumor with CRTC1::TRIM11 fusion and prominent epidermal involvement: a case report. J Cutan Pathol. 2022;49(12):1025‐1030. doi:10.1111/cup.14287 35751643 PMC10086857

[22] Bui CM , Chaum M , Balzer B . A case of digital cutaneous melanocytic tumor with CRTC1::TRIM11 fusion. Cureus. 2022;14:e33179. doi:10.7759/cureus.33179 36726909 PMC9886156

[23] Shang M , Weng L , Xu G , et al. TRIM11 suppresses ferritinophagy and gemcitabine sensitivity through UBE2N/TAX1BP1 signaling in pancreatic ductal adenocarcinoma. J Cell Physiol. 2021;236(10):6868‐6883. doi:10.1002/jcp.30346 33629745

[24] Buchberger DS , Videtic GMM . Stereotactic body radiotherapy for the management of early‐stage non‐small‐cell lung cancer: a clinical overview. JCO Oncol Pract. 2023;19(5):239‐249. doi:10.1200/OP.22.00475 36800644

[25] Pezzuto A , Cappuzzo F , D'Arcangelo M , et al. Prognostic value of p16 protein in patients with surgically treated non‐small cell lung cancer; relationship with Ki‐67 and PD‐L1. Anticancer Res. 2020;40(2):983‐990. doi:10.21873/anticanres.14032 32014943

[26] Li M , Zhao S , Lopez G , et al. Mean platelet volume, thrombocytosis, and survival in non‐small cell lung cancer patients treated with first‐line pembrolizumab alone or with chemotherapy. Cancer Immunol Immunother. 2023;72(7):2067‐2074. doi:10.1007/s00262-023-03392-9 36795122 PMC10991400

[27] Çetin İ , Topçul M . Antiproliferative effects of EGFR inhibitor cetuximab and PARP inhibitor combination on non‐small cell lung cancer cell line A549 and cervical cancer cell line HeLa. Cell Mol Biol (Noisy‐le‐Grand). 2022;68(8):47‐51. doi:10.14715/cmb/2022.68.8.8 36800840

[28] Zhao Z , Deng J , Lu M , et al. TRIM11, a new target of p53, facilitates the migration and invasion of nasopharyngeal carcinoma cells. Mol Biol Rep. 2023;50(1):731‐737. doi:10.1007/s11033-022-07833-z 36376537 PMC9884187

[29] Jiaqi L , Siqing H , Qin W , Di Z , Bei Z , Jialin Y . Andrographolide promoted ferroptosis to repress the development of non‐small cell lung cancer through activation of the mitochondrial dysfunction. Phytomedicine. 2023;109:154601. doi:10.1016/j.phymed.2022.154601 36610134

[30] Zhang C , Lu X , Liu X , et al. Carbonic anhydrase IX controls vulnerability to ferroptosis in gefitinib‐resistant lung cancer. Oxid Med Cell Longev. 2023;2023:1367938. doi:10.1155/2023/1367938 36760347 PMC9904911

[31] Lu X , Kang N , Ling X , Pan M , Du W , Gao S . MiR‐27a‐3p promotes non‐small cell lung cancer through SLC7A11‐mediated‐ferroptosis. Front Oncol. 2021;11:759346. doi:10.3389/fonc.2021.759346 34722314 PMC8548660

[32] Luo L , Xu G . Fascaplysin induces apoptosis and ferroptosis, and enhances anti‐PD‐1 immunotherapy in non‐small cell lung cancer (NSCLC) by promoting PD‐L1 expression. Int J Mol Sci. 2022;23(22):13774. doi:10.3390/ijms232213774 36430250 PMC9699238

[33] Wang L , Fu H , Song L , et al. Overcoming AZD9291 resistance and metastasis of NSCLC via ferroptosis and multitarget interference by nanocatalytic sensitizer plus AHP‐DRI‐12. Small. 2023;19(4):e2204133. doi:10.1002/smll.202204133 36420659

[34] Miao Y , Chen Y , Xue F , et al. Contribution of ferroptosis and GPX4's dual functions to osteoarthritis progression. EBioMedicine. 2022;76:103847. doi:10.1016/j.ebiom.2022.103847 35101656 PMC8822178

[35] Seibt TM , Proneth B , Conrad M . Role of GPX4 in ferroptosis and its pharmacological implication. Free Radic Biol Med. 2019;133:144‐152. doi:10.1016/j.freeradbiomed.2018.09.014 30219704

[36] Yao Y , Chen Z , Zhang H , et al. Selenium‐GPX4 axis protects follicular helper T cells from ferroptosis. Nat Immunol. 2021;22(9):1127‐1139. doi:10.1038/s41590-021-00996-0 34413521

[37] Chen C , Wang D , Yu Y , et al. Legumain promotes tubular ferroptosis by facilitating chaperone‐mediated autophagy of GPX4 in AKI. Cell Death Dis. 2021;12(1):65. doi:10.1038/s41419-020-03362-4 33431801 PMC7801434

[38] Zhang W , Lu J , Wang Y , et al. Canagliflozin attenuates lipotoxicity in cardiomyocytes by inhibiting inflammation and ferroptosis through activating AMPK pathway. Int J Mol Sci. 2023;24(1):858. doi:10.3390/ijms24010858 36614295 PMC9821072

[39] Wang X , Chen X , Zhou W , et al. Ferroptosis is essential for diabetic cardiomyopathy and is prevented by sulforaphane via AMPK/NRF2 pathways. Acta Pharm Sin B. 2022;12(2):708‐722. doi:10.1016/j.apsb.2021.10.005 35256941 PMC8897044

[40] Zhao Y , Li M , Yao X , et al. HCAR1/MCT1 regulates tumor ferroptosis through the lactate‐mediated AMPK‐SCD1 activity and its therapeutic implications. Cell Rep. 2020;33(10):108487. doi:10.1016/j.celrep.2020.108487 33296645

[41] Lee H , Zandkarimi F , Zhang Y , et al. Energy‐stress‐mediated AMPK activation inhibits ferroptosis. Nat Cell Biol. 2020;22(2):225‐234. doi:10.1038/s41556-020-0461-8 32029897 PMC7008777

[42] Liu Y , Xu Y , Wang F , et al. Inhibition of AMPK activity by TRIM11 facilitates cell survival of hepatocellular carcinoma under metabolic stress. Clin Transl Med. 2021;11(12):e617. doi:10.1002/ctm2.617 34919347 PMC8679837

